# From digestion and absorption to innate immunity and health care: water and food intake may contribute to IL-22 in ILC3-dependent mucosal immunity in the jejunum

**DOI:** 10.1186/s12576-021-00817-x

**Published:** 2021-10-12

**Authors:** Tomomi Watanabe-Asaka, Moyuru Hayashi, Daisuke Maejima, Yoshiko Kawai, Toshio Ohhashi

**Affiliations:** 1 Department of Innovation of Medical and Health Sciences Research, Shinshu University School of Medicine, 3-1-1 Asahi, Matsumoto, 390-8621 Japan; 2 Division of Physiology, Faculty of Medicine, Tohoku Medical and Pharmaceutical University, Sendai, Japan

**Keywords:** Jejunum, Food absorption, IL-22, ILCs 3, Mucosal immunity, Health care

## Abstract

In this review, with our current studies we demonstrated medical evidence that water and food intake are useful for IL-22-related mucosal immunity-dependent maintenance of health care. The traditional Japanese health care practices recommend daily consumption of suitable volume of water. However, immunological mechanisms that support of the traditional practices are still unsolved. We focused on type 3 innate lymphoid cells (ILC3s), because the ILC3s are mainly housed in the lamina propria of the jejunum. IL-22 released from the ILC3 is transported through mesenteric lymph in collaboration with the albumin-mediated movement of consumed water. Thus, water intake-mediated upregulation of IL-22-dependent mucosal immunity contributes to the traditional Japanese health care practices. We also reviewed current studies that food intake-mediated increase in VIP-dependent neuronal activity in the small intestine and the food intake included with tryptophan-derived metabolites may accelerate the IL-22 in ILC3s-dependent mucosal immunity and then contribute in keeping health care.

## Introduction

The traditional Japanese health care system, known since the Edo period, recommends that a suitable volume of water be consumed every day [1]. In addition, the Japanese eating has a characteristic property with taking miso soup at every time. However, the physiological and immunological mechanisms in support of these traditional practice and eating are still lacking. Meanwhile, the jejunal microcirculation compared with those in other organs, has specific properties such as movement of large amounts of albumin from the venular walls to tissues and its related higher tissue osmotic pressure at the venular side [2, 3]. Jejunum-originated mesenteric lymph flow is also known to be much more than those in other organs. Consistent with these properties, the mesenteric collecting lymph vessels show heart-like spontaneous contractions which contribute to transport actively large amount of lymph [4–6].

Innate lymphoid cells (ILCs), especially type 3 ILCs (ILC3s) are currently known to be housed in the small intestine and contribute to the mucosal immunity and barrier mechanisms in the jejunum [7]. In this review, we focus on the physiological and immunological roles of ILC3s-derived IL-22 in the digestion and absorption function in the jejunum for health care.

## ILC3s are mainly localized in the lamina propria of the upper part of the rat jejunum

ILCs are one of the most recently identified cell populations that contributed to innate immune responses. ILCs lack the antigen-specific receptors that characterize adaptive T and B lymphocytes, but are exquisitely attuned to the local tissue environment and can rapidly respond to tissue damage or pathogen threats [7]. Several subgroups of ILCs have been identified that are best characterized by their effector functions, which include cytokine secretion and cytotoxic killer potential. These subgroups include the interferon-γ-expressing group 1 ILCs, which include natural killer (NK) cells; the IL-5 and IL-13-expressing ILC2s; and the heterogeneous group 3 ILCs, which include lymphoid-tissue-inducer cells and IL-17 and IL-22-expressing cells [7–11]. These mature subsets are distinguished by the characteristic transcription factors T-bet for ILC1s, GATA-3 for ILC2s, and RORγt for ILC3s. Although ILCs reside in most tissues, they serve prominent roles in barrier tissues such as the skin, intestine, and lungs. ILCs respond more rapidly than T lymphocytes to tissue damage mediated by microbial infection. Especially, ILC2 and ILC3 cells express receptors for various inflammatory mediators. These cells integrate multiple signals that arise simultaneously within the tissue, and by their elaboration on mediators, they can function as immunological first responders to repel parasites, and bacterial and fungal infection. Their functions seem to coordinate subsequent adaptive immune response. The ILC2 and ILC3 also promote the tissue-repair process to restore to tissue homeostasis [7–11].

On the other hand, with a more comprehensive and integrated view of data on ILCs tissue distribution, most of ILC 3 cells in the body were distributed in the lamina propria and intraepithelial compartment of small intestine, being more than those tissues of the colon [12]. However, there are no data on the difference of ILC3 distribution between the jejunum and ileum. In addition, there are several questions what physiological and immunological meanings underlie the different distribution of ILC3 cells between the jejunum and ileum. Taken together, we investigated the distribution and dynamics of ILC3s in the lamina propria of the jejunal and ileal walls, mesenteric lymph nodes, and the mesenteric lymph of rats using flow cytometry and quantitative RT-PCR [13].

**Fig. 1:**
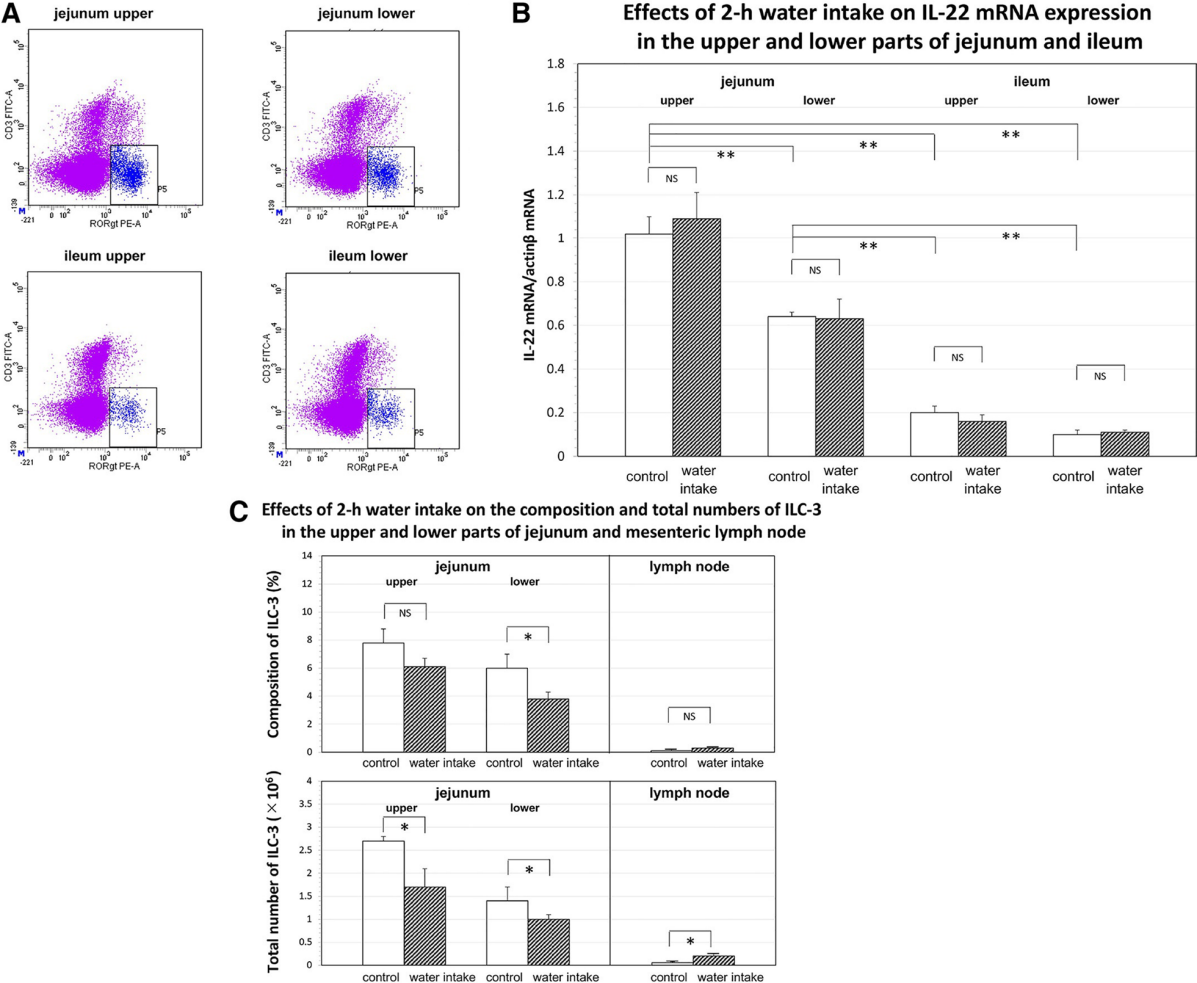
**A** Representative flow cytometry recordings of RAR-related orphan receptor-γ (RORγt)-positive cells (blue) in the lamina propria of upper and lower parts of jejunum and ileum in the control. The ordinate and abscissa of each panel show fluorescent intensity of CD3-FITC and RORγt-PE, respectively. For flow cytometry analysis, the gating strategy to isolate ILC3s from the collected cells was as follows: (1) isolation of the live cells, (2) gating and isolation of CD-45 positive cells, and then (3) gating and isolation of CD-45R- and CD-3-negative and RORγt-positive cells. **B** Effects of 2-h water intake on expression of IL-22 mRNA in the lamina propria of upper and lower parts of jejunum and ileum. Expression levels of IL-22 mRNA were normalized relative to those of β-actin (ordinate, *n* = 5). Open and dappled bars show control and treatment with 2-h water intake, respectively. **p < 0.01; *NS* not significant. **C** Effects of 2-h water intake on composition (top) and total number (bottom) of innate lymphoid cells 3 (ILC-3; *n* = 5) in upper and lower parts of the jejunum and in mesenteric lymph node (the ordinate). Open and dappled bars show control and treatment with 2-h water intake, respectively. *p < 0.05 (with permission of ref. [13])

Figure 1A (with permission of ref. [13]) shows representative flow cytometry data on RORγt-positive ILC3. There was a marked heterogeneity in the distribution of ILC3s between jejunum and ileum. Thus, ILC3s were localized mainly in the lamina propria of the upper part of the jejunum. The number of ILC3s was 8.4 ± 1.0% of the total lymphocytes in the lamina propria. Consistent with the flow cytometry data on ILC3s, the expression of IL-22 mRNA showed a marked heterogeneity between the jejunum and ileum (Fig. 1B, with permission of ref. [13]). Thus, the expression of IL-22 mRNA was the highest in the upper part of jejunum. No significant effect of 2-h water intake was observed on the IL-22 mRNA expression in the jejunum and ileum. On the other hand, 2-h water intake resulted in a significant decrease in the total number of ILC3s in both upper and lower parts of the jejunum (Fig. 1C, with permission of ref. [13]). In contrast, the total number of ILC3s in mesenteric lymph nodes was significantly increased by 2-h water intake. No significant presence of ILC3s in the mesenteric lymph was observed under both control or water intake conditions. In contrast, Hokari et al. [14] recently demonstrated that the indomethacin-induced inflammation increased the movement of ILC3s through the mesenteric lymph vessels. Next, to evaluate the effects of water intake on the activity of ILC3s in the lamina propria of rat jejunum, we measured the concentration of IL-22 in the mesenteric lymph collected over 60 min periods using the mouse/rat IL-22 ELISA kits. The total flux of IL-22 through the mesenteric lymph vessel, calculated by multiplying the concentration of IL-22 by the lymph volume, significantly increased at 1 h after the water intake. This was due to the accelerated jejunum-originated mesenteric lymph flow during the 1-h period after the water intake. The concentration of IL-22 in the control mesenteric lymph was 299.5 ± 46.9 pg/ml (*n* = 5), which was significantly higher than the values obtained in the arterial (66.1 ± 8.4 pg/ml, *n* = 5) and venous blood (60.4 ± 7.2 pg/ml, *n* = 5).

In conclusion, the localization of ILC3s and the expression of IL-22 mRNA were highest in the small intestine, especially the upper part of jejunum. The concentration of IL-22 in the mesenteric lymph was approximately 7- to 8-fold higher than those in the arterial and venous blood. The difference of IL-22 concentration between the mesenteric lymph and the blood may be related to the presence of IL-22 binding protein (IL-22 BP, also known as IL-22RA 2) [15–17]. Further investigation will be needed to clarify the mechanisms for producing the difference of concentration of IL-22 between mesenteric lymph and blood.

Next question is how these findings are related to the physiological and immunological function of the jejunum and mesenteric lymph for food digestion and absorption, and health care.

## Water intake accelerates mesenteric lymph flow and the total flux of albumin, long-chain fatty acids, and IL-22 in rats

It is well known that the digestion of dietary lipids, carbohydrates, and proteins is initiated in the lumen of the duodenum and the proximal jejunum and is completed at the glycocalyx and microvillus plasma membrane of the jejunal absorptive cells. In addition, the microcirculation of the small intestine, especially the jejunum, has specific properties so that the movement of larger amounts of plasma proteins into the mucosal interstitial space through venular walls may be advantageous for the movement of protein-bound substances to mesenteric lymph vessels. The existence of specialized lymph capillaries, lacteal vessels in the jejunal villi, may be consistent with these functional properties of microcirculation and lymphatic circulation in the jejunum [3, 18–20]. In agreement with these specific properties of microcirculation in the jejunum, the jejunal-originated collecting lymph vessels in human, cow, and rat exhibit heart-like spontaneous contractions which contribute to transport actively large amounts of mesenteric lymph actively [4–6]. The activity of heart-like spontaneous contractions is regulated by aminergic sympathetic nerve fibers [4–6].

We next focused on the roles of ILC3s-mediated IL-22 in the physiological and immunological functions related with the water intake and health care [13]. In vivo experiments were conducted on rats [13]. To collect the lymph from the jejunal-originated mesenteric lymph vessel, the abdomen was opened by cutting the midline, and mesenteric adipose and connective tissues were removed to expose the mesenteric lymph node and the efferent lymph vessel. A small polyethylene catheter was inserted centrifugally into the efferent lymph vessel. To evaluate the effects of water intake on the mesenteric lymph flow and the concentrations of cells, albumin, long-chain fatty acids, and IL-22 in the lymph, an intragastric administration of distilled water (3 ml) was performed by inserting a needle catheter through the mouth to the stomach. The reason why the distilled water used in the experiments was related to exclude the opposite effects of physiological saline solution (PSS)-derived ionic osmotic pressure on the albumin-mediated tissue colloid osmotic pressure-dependent movement of water-soluble substances in the lamina propria of jejunal villi. The greatest amount of lymph fluid was collected in the first 15 min after the intragastric administration of distilled water. Next, the effects of water intake on the concentration of albumin in the lymph and the total flux of albumin through the lymph vessel were evaluated. The concentration of albumin was significantly decreased at 1 h after water intake. In contrast, the total flux of albumin through the lymph vessel was significantly increased only for 1 h after intake due to the significant increase in the mesenteric lymph flow. Surprisingly, the agreement with the findings for albumin, the concentrations of long-chain fatty acids in the lymph were significantly decreased at 1 h after water intake and the total flux of long-chain fatty acids through the lymph vessel was significantly increased at 1 h after water intake. Interestingly, the concentration of long-chain fatty acids in the mesenteric lymph under control conditions of overnight fasting was approximately seven times higher than those in the arterial and venous blood. The total flux of IL-22 through the lymph vessel also was significantly increased at 1 h after water intake.

In conclusion, the higher permeability of albumin-mediated transport of water-soluble substances in mesenteric lymph vessels of the jejunum may have a large impact on the classical concept suggesting that water-soluble small molecules travel to the liver via the portal vein. The long-chain fatty acids in the lymph were transferred from the jejunal villi to mesenteric lymph by water intake, suggesting that dietary intake of long-chain fatty acids may be stored into the lamina propria of jejunal villi. The finding may support the evidence that the mesenteric lymph was named “white blood” by Hippocrates in ancient Greece [21]. ILC3 is mainly housed in the lamina propria of the jejunum. In addition, IL-22 released from the ILC3s is transported through mesenteric lymph in collaboration with the albumin-mediated movement of consumed water. Thus, the water intake-mediated and highly exuded of albumin-dependent movement of IL-22 from the mesenteric lymph to blood may contribute to the traditional Japanese health care system with the IL-22-dependent maintaining and promoting the innate immunity in the body. In fact, two interesting papers related with the IL-22-dependent innate immunity in the body have been recently published in *Nature* and *Nature Medicine* [22, 23]. The cytokine IL-22, produced by ILC3s, is an important regulator of the DNA damage response (DDR) machinery in intestinal epithelial stem cells. Using a new mouse model that enables sporadic inactivation of the IL-22 receptor in colon epithelial stem cells, they represented that IL-22 is required for effective initiation of the DDR following DNA damage. Stem cells deprived of IL-22 signals and exposed to carcinogens escaped DDR-controlled apoptosis, contained more mutations and were more likely to give rise to colon cancer [22]. Modifying the gut microbiota, altering bile acid metabolism and/or increasing IL-22 levels may be of value for the treatment of polycystic ovary syndrome [23]. However, further investigation will be needed in the future to elucidate the detailed critical roles of IL-22 in the maintaining and promoting the innate immunity for keeping health care.

## Water intake releases ATP in rat jejunum, resulting in upregulation the IL-22 mRNA expression in ILC3s

ATP has been known to play a key controller in the Treg and Th17-mediated mucosal immunity in the small intestine [24–26]. To verify the physiological and immunological roles of ATP in rat jejunal villi, we established a myofibroblast cell line in the rat small intestine to examine the effects of shear stress stimulation on the culture cells [27], and then identified the effects of water intake-mediated shear stress stimulation in the jejunal villi, because ATP is released by shear stress stimulation from blood and lymphatic endothelial cells via the activation of the cell surface *F*_1_/*F*_0_ ATP synthase [28–30]. ATP release was significantly increased when shear stress stimulation (1 dyn/cm^2^) was applied to the cultured myofibroblast cells. ATP also increased the immunocytochemical expression of podoplanin and upregulated the podoplanin mRNA expression in the cultured human intestinal epithelial cells. Next, we investigated whether ATP is involved in the immunohistochemical expression of podoplanin in the jejunal villi through the activation of water intake in vivo rat experiments. As expected, water intake caused a marked increase in podoplanin expression in the interepithelial layers and lamina propria of jejunal villi. ATP also induced upregulation of podoplanin mRNA expression in the isolated cells from the jejunal villi in a dose-dependent manner.

**Fig. 2:**
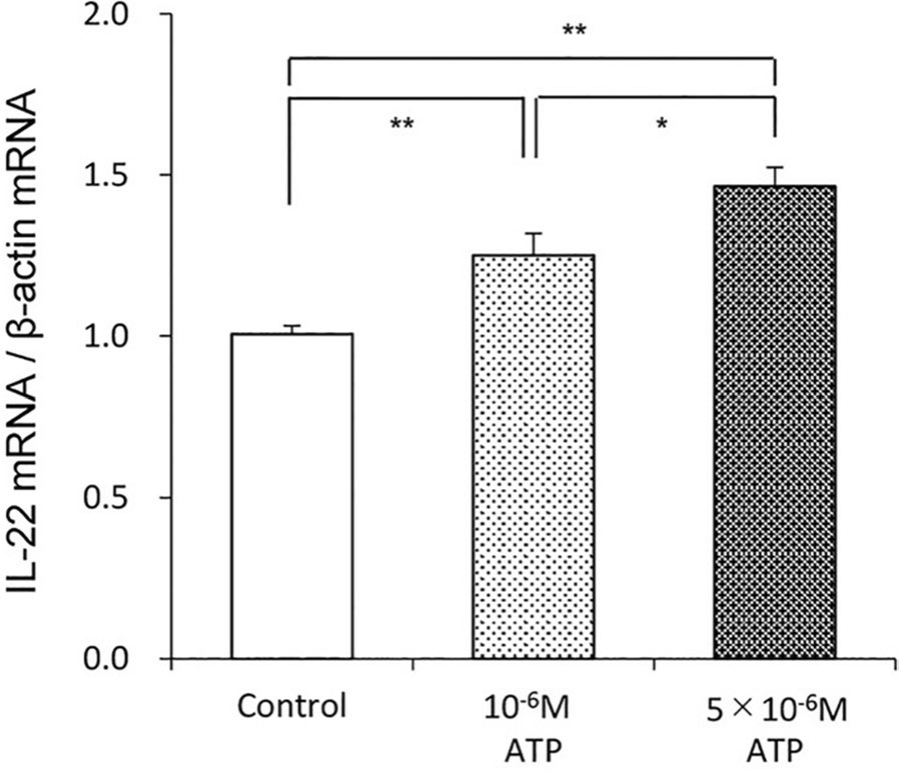
ATP (10^–6^ M to 5 × 10^–6^ M) dose-dependently increased IL-22 mRNA expression in non-T, no-B, and RORγt-positive cells isolated from the lamina propria of jejunal villi. *p < 0.05, **p < 0.01 (with permission of ref. [27])

To evaluate the immunological roles of ATP in ILC3s localized in the jejunal villi, we firstly isolated the ILC3s as non-T, non-B, and RORγt-positive cells by flow cytometry. To confirm the RORγt-positive cells as ILC3s, we examined the presence of CD127 marker on CD45- and RORγt-positive cells. Thus, most of CD45- and RORγt-positive cells expressed CD127. The IL-22 mRNA expression in the CD45-positive and CD3-negative lymphocytes was increased by exogenous ATP in a dose-dependent manner (Fig. 2, with permission of ref. [27]). The immunohistochemical expression of IL-22 in the lamina propria of jejunal villi was significantly upregulated by water intake and this upregulation was significantly suppressed by the pretreatment with intravenous administration of a non-selective purinergic receptor antagonist, suramin.

From the above, a great bulk flow-dependent mechanical force due to water intake accelerates ATP release in rat jejunal villi. And this water intake-mediated ATP release in the jejunal villi upregulates the podoplanin and IL-22 immunohistochemical expression in the interepithelial layers and lamina propria of the jejunum. Water intake also upregulates the IL-22 mRNA expression in ILC3s in the lamina propria of jejunal villi, which may contribute to the regulation of mucosal immunity in the jejunum. In addition, water intake-mediated upregulation of podoplanin in the jejunal villi maintains physiologically higher tissue colloid osmotic pressure in the jejunal interstitial space, resulting in the production of large amount of jejunal mesenteric lymph flow.

## Water intake releases serotonin in rat jejunum, resulting in upregulation of the IL-22 mRNA expression in ILC3

Serotonin, a vasoactive substance, is a neurotransmitter and hormone that contributes to the regulation of various physiological functions in the central nervous system and their respective organ systems. Peripheral serotonin is predominantly produced by enterochromaffin cells in the small intestine [31–33]. Furthermore, serotonin in the blood is taken up and stored in platelets [34]. We aimed to evaluate the roles of water intake on the release of serotonin from the enterochromaffin cells and to clarify the effects of water intake-dependent release of serotonin on IL-22 mRNA and immunoreactivity of IL-22 in rat jejunal villi. Additionally, we evaluated the physiological roles of blood serotonin on lymph formation in the jejunal microcirculation, along with the effects of water intake on changes in the concentration of platelets in the blood [35].

**Fig. 3:**
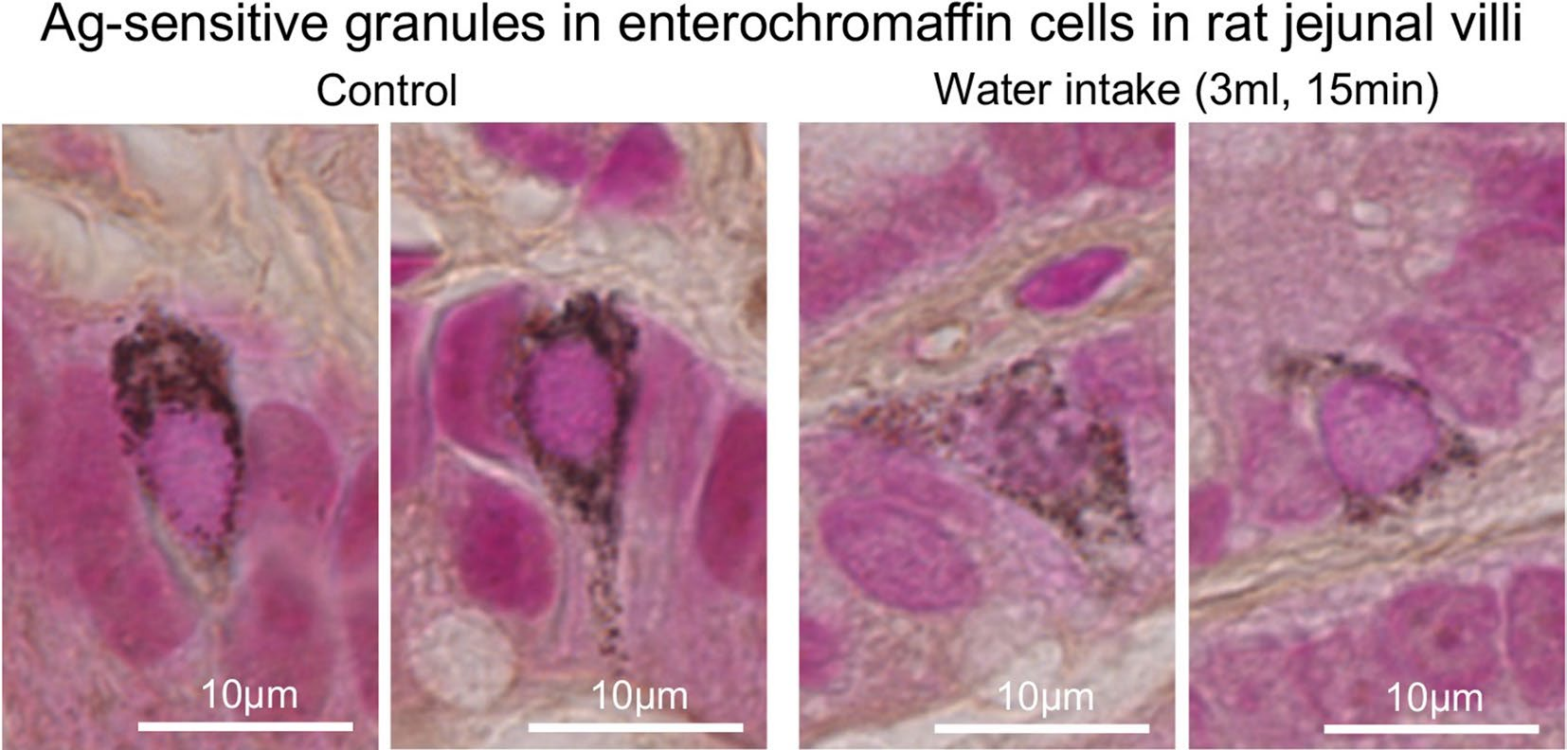
Representative photomicrographs for Ag-sensitive black-colored granules in the enterochromaffin cells in rat jejunal villi stained with Grimelius staining method with AgNO_3_ (with permission of ref. [35])

**Fig. 4:**
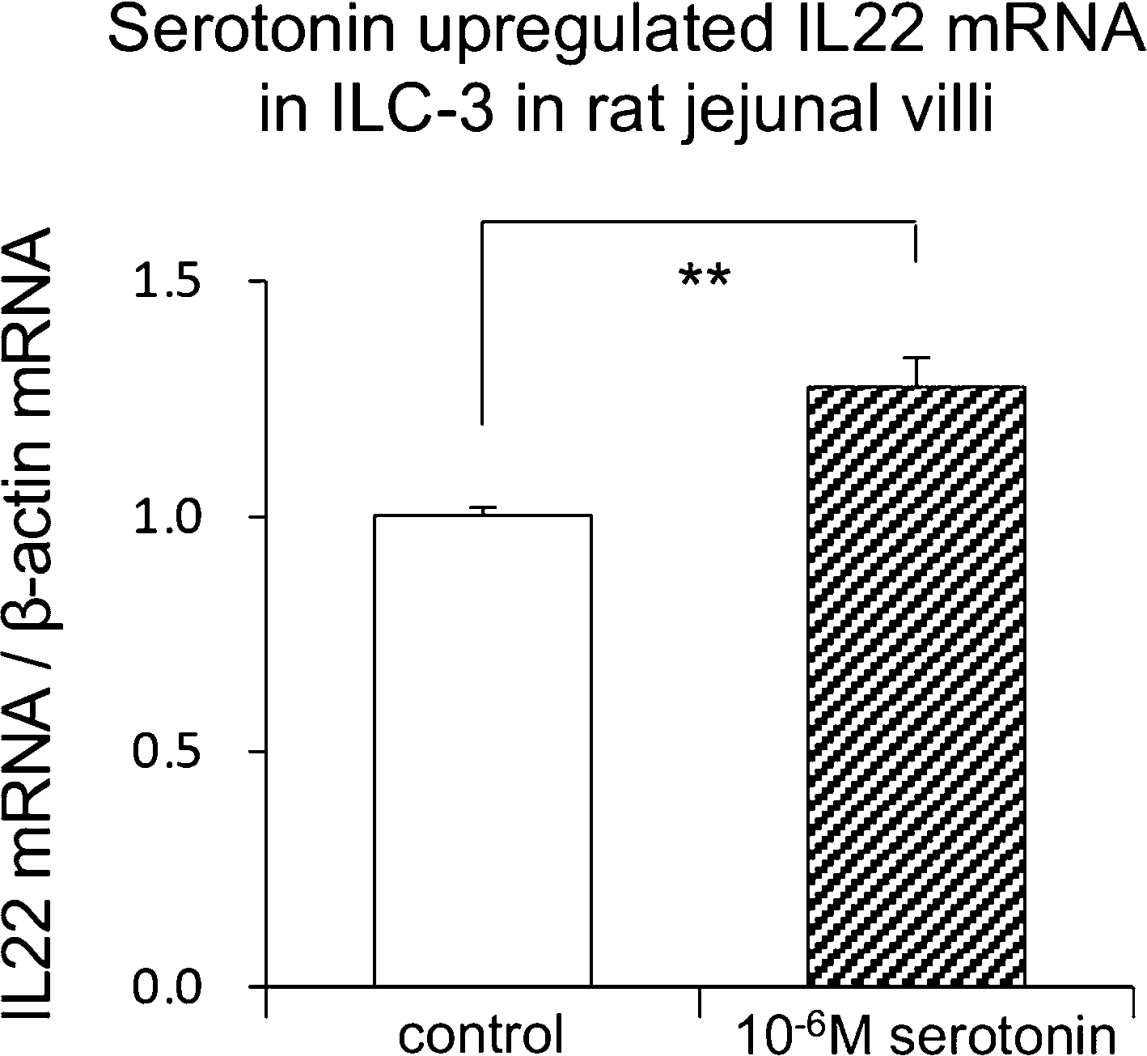
The effect of serotonin on IL-22 mRNA expression in non-T, non-B, and RORγt-positive cells isolated from the lamina propria of jejunal villi. **p < 0.01 (with permission of ref. [35])

To determine whether water intake induces serotonin release in rat jejunal villi, we investigated the serotonin immunoreactivity in the jejunal villi. Water intake significantly increased the serotonin immunoreactivity in the lamina propria of jejunal villi. In addition, the Ag-sensitive black-colored granules in the enterochromaffin cells stained by Grimelius method in the jejunal villi clearly disappeared after water intake (Fig. 3, with permission of ref. [35]). Water intake brought significant increase of the serotonin concentration and the total flux of serotonin in the rat portal vein at the first 15 min after water intake. The increased concentration and total flux of serotonin returned to the original control condition by 30 min after water intake. However, water intake did not induce significant change in the concentration of serotonin in mesenteric lymph derived from the jejunum because the serotonin may be bound to larger molecules such as fatty acids in the lamina propria [35]. However, water intake significantly increased the mesenteric lymph volume within 15 min of water intake, which was consistent with our previously demonstrated data [13]. Intravenous administration of serotonin increased the concentrations of albumin and IL-22 in the jejunal-originated mesenteric lymph within the 60 min after the administration of serotonin. Next, to evaluate the critical roles of serotonin in the ILC3s-dependent regulation of mucosal immunity in the jejunal villi, we investigated the effects of serotonin on the IL-22 mRNA expression in ILC3s in rat jejunal villi using the CD45-, and CD127-positive lymphocytes in the lamina propria. Serotonin induced the upregulation of IL-22 mRNA expression in ILC3s in the jejunal villi (Fig. 4, with permission of ref. [35]). To confirm the serotonin-mediated upregulation of IL-22 mRNA expression in ILC3s, we further investigated the effects of water intake on the IL-22 immunoreactivity in the jejunal villi. The water intake for 45 min significantly increased the immunoreactivity of IL-22 in the jejunal villi. These results indicated that water intake released serotonin from enterochromaffin cells in rat jejunal villi and that serotonin was transported mainly through the portal vein. Intravenous administration of serotonin increased mesenteric lymph volume and concentrations of albumin and IL-22 in the lymph, suggesting that serotonin in blood physiologically regulates the mesenteric lymph formation. Also, serotonin regulates mucosal immunity by the upregulation of IL-22 mRNA expression in ILC3s in jejunal villi.

## The neuropeptide, vasoactive intestinal peptide (VIP), regulates ILC3s-dependent mucosal immunity with food intake

VIP is colocalized with acetylcholine in the nerve terminal of cholinergic parasympathetic nerve fibers [36] innervated into the small intestine and causes the vasodilation in the microcirculation of the small intestine to assist the autonomic nervous function of parasympathetic nerve fibers [36]. In addition, the jejunal-originated mesenteric lymph vessels are innervated with autonomic nerve fibers included VIP as a chemical transmitter [36], and physiologically regulates the lymph transport in the mesenteric lymph vessels. Two very impressive papers have recently been published showing that VIP regulates ILC3s-dependent mucosal immunity in response to food intake [37, 38]. The function of ILC3s is not constant throughout the day, but instead oscillates between active and resting phases. Coordinate responsiveness of ILC3s in the small intestine was dependent on the food-induced expression of the neuropeptide VIP. Intestinal ILC3s had high expression of the G protein-coupled receptor VIP receptor 2 (VIPR2), and its activation by VIP markedly enhanced the production of IL-22 and the barrier function of the epithelium in the small intestine. Conversely, deficiency in the VIPR2-mediated signaling led to impaired production of IL-22 by ILC3s and increased gut susceptibility to inflammation-induced injury. Thus, intrinsic cellular rhythm acted synergistically with the cyclic patterns of food intake to drive the production of IL-22 and synchronize protection of the intestinal epithelium through a VIP-VIPR2 pathway in ILC3s [37]. However, it remains unclear how the intestine coordinates physiological and immune responses to food consumption to optimize nutrient uptake while maintaining barrier functions remains unclear. In contrast, Talbot et al. [38] showed in mice how a gut neuronal signal triggered by food intake is integrated with intestinal antimicrobial and metabolic responses that are controlled by ILC3s. During food consumption, the activation of VIP-operating neurons thus enhances the growth of segmented filamentous bacteria associated with the epithelium of small intestine, and increases lipid absorption. Indeed, there was more VIP in portal vein after 6 h of free-feeding than after 6 h fasting [38]. However, VIPR2-dependent inhibition of IL-22 production in ILC3s after food intake was proved in the mice experiments [38]. Talbot’s finding that food intake inhibited the production of IL-22 in ILC3s [38] may sound opposite to our results [12, 27, 35] that water intake upregulated the IL-22 mRNA expression in ILC3s in rat jejunal villi which is compatible with the former finding [37]. The difference in contribution of VIP–VIPR2 interactions between food and water intake may be related to the opposite findings. Further investigation will be needed in the future to clarify the mechanisms to produce the controversial findings.

## Tryptophan-derived metabolites contribute to mucosal immunity for health care by IL-22 through the activation of aryl hydrocarbon receptor (AhR) on ILC 3

The AhR, a basic helix–loop–helix protein, is a member of the Per-AhR-nuclear translocator-Sim superfamily of proteins [39]. AhR is a cytosolic transcription factor that is ubiquitously expressed in vertebrate cells and can be activated by a wide variety of natural and synthetic ligands, including environmental, tryptophan-derived dietary, and endogenous aromatic compounds [40]. In its inactive state, AhR resides in the cytosol bound to several cochaperones such as heat shock protein 90. Upon ligand binding, chaperones are released and AhR moves to the nucleus, where it binds to its dimerization partner, AhR-nuclear translocator, thus initiating the transcription of a variety of target genes [41]. In recent years, AhR has gained attention because it represents an important role in linking the environment and immunity. Thus, an activation of AhR by the microbiota in the intestinal tract is clarified to be essential for promoting IL-22 production in ILC3s [42]. Specially, tryptophan-derived catabolites generated via the microbiota metabolism are involved in mucosal immune responses through AhR modulation [42]. Currently, the breakdown products of cruciferous vegetables such as cauliflower and Brussels sprout are ligands for AhR, and AhR-mediated signaling in ILC3s and γδ T cells controlled their production of IL-22 [42]. Thus, AhR signaling, in turn, ensures on-demand production of IL-22 by innate lymphocytes directly regulating components of the DNA damage response in epithelial stem cells [42]. In conclusion, eating vegetables such as cauliflower and broccoli may accelerate IL-22-dependent mucosal immunity for health care.

## Conclusion and future perspectives

In this review, we summarized the roles of water intake-dependent IL-22 in ILC3s in rat jejunal villi in contributing to traditional Japanese health care. We found that water intake increased the jejunum-originated mesenteric lymph flow and total flux of IL-22 through the lymph vessels. Water intake accelerated the ATP release from myofibroblast cells in the jejunum. ATP upregulated IL-22 mRNA in ILC3s in the lamina propria of jejunal villi. ATP also induced the podoplanin expression in the interepithelial layers and lamina propria of jejunal villi which contributed to keep the higher level in colloid osmotic pressure in the tissues, resulting in water intake-mediated increase in the movement of IL-22 through the mesenteric lymph vessels. Water intake released serotonin from enterochromaffin cells in the jejunal villi. Serotonin upregulated IL-22 mRNA in ILC3s. Based on these findings, water intake-mediated IL-22-dependent mucosal immunity contributes, in part, to the traditional Japanese health care practices.

**Fig. 5:**
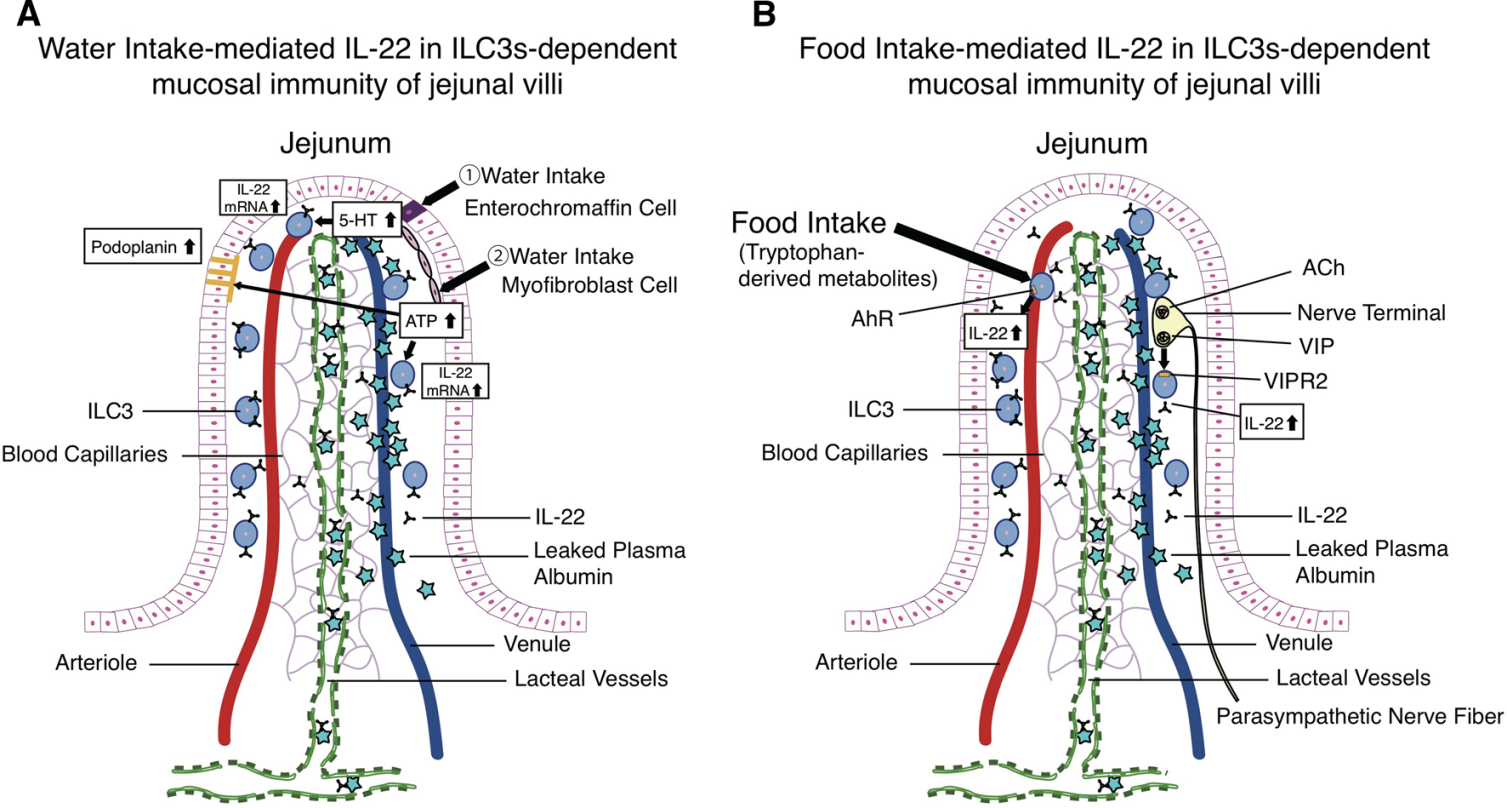
**A** A graphical abstract summarized water intake-mediated IL-22 in ILC3s-dependent mucosal immunity in jejunal villi. **B** A graphical abstract summarized food intake (the activation of VIP-VIPR2 or Ahr)-mediated IL-22 in ILC3s-dependent mucosal immunity

Living organisms including humans take in nutrients through the food ingestion, and the nutrient uptake is conducted through the jejunal microenvironment where substances are directly transferred from outside to the internal body. The maintenance of the immune system in the jejunal microenvironment is especially important to maintain health care in all living organisms. The fact that some chemical compounds from food can activate such IL-22 in ILC3s-dependent innate immunity suggests a necessary part of the perfect balance to maintain of our health. Figure 5 demonstrates water (A) and food (B) intake-dependent IL-22 in ILC3s-mediated mucosal immunity in the jejunum.

## Data Availability

All relevant data are available from the corresponding author on request.

## Abbreviations

AhR: Aryl hydrocarbon receptor
DDR: DNA damage response
ELISA: Enzyme-linked immunosorbent assay
IL-22: Interleukin 22
IL-22 BP: IL-22 binding protein
ILC3s: Type 3 innate lymphoid cells
NK cell: Natural killer cell
PSS: Physiological saline solution
RT-PCR: Reverse transcription-polymerase chain reaction
VIP: Vasoactive intestinal peptide
